# Path Model of Risk Factors for Age at Primary Tooth Eruption: A Cohort Study of Preterm and Term Infants

**DOI:** 10.3390/ijerph22121837

**Published:** 2025-12-09

**Authors:** Bianca S. Tavares, Jéssica M. Bittencourt, Joana Ramos-Jorge, Saul M. Paiva, Jhonathan Lopes-Silva, Cristiane B. Bendo

**Affiliations:** 1 Department of Pediatric Dentistry, School of Dentistry, Universidade Federal de Minas Gerais, Av. Antonio Carlos 6627, Belo Horizonte 31270-901, MG, Brazil; biancaspurit@ufmg.br (B.S.T.); jrj2008@ufmg.br (J.R.-J.); smpaiva@ufmg.br (S.M.P.); crysbendo@ufmg.br (C.B.B.); 2 Faculty of Dentistry, Centro Universitário Arnaldo Janssen, UNIARNALDO, Praça Arnaldo Janssen, 200-Funcionários, Belo Horizonte 30130-066, MG, Brazil; jhonathan.silva@profarnaldo.com.br

**Keywords:** tooth, deciduous, infant, premature birth

## Abstract

Several factors have been associated with delayed eruption of primary teeth. Thus, the objective of the study was to test a path model of the direct and indirect birth-related risk factors influencing the age of first primary tooth eruption in infants. Cohort study with 43 preterm and 48 full-term infants aged at least four months. Infants were monitored monthly to verify the chronology of eruption of the first primary tooth. Mothers responded to sociodemographic and health behavior questionnaire. Principal Component Analysis and path analysis were performed. Two models were constructed: chronological and corrected age of tooth eruption. Model using chronological age of tooth eruption demonstrated that preterm infants had an increased risk of having later tooth eruption compared to those born at term ([β] = 0.888; *p* < 0.001). Indirect associations were found between socioeconomic and health conditions with the age of tooth eruption, mediated by gestational age. The same direct and indirect associations were also found for corrected age, with differences only in β values. It is concluded that preterm infants exhibited higher risk of delayed tooth eruption compared to full-term infants, considering both chronological and corrected age. Gestational age mediated the association between socioeconomic and health conditions with the age of tooth eruption.

## 1. Introduction

The development of the human deciduous dentition begins at the end of the fifth week of gestation [1,2]. Before eruption, the tooth completes crown formation and mineralization, while the root continues to develop for approximately 18 months after eruption [1,2]. The ages at which primary teeth erupt are very important for children’s craniofacial growth and development [1].

Several factors have been associated with delayed eruption of primary teeth, such as malnutrition, endocrine disorders, genetic factors, congenital syndromes, and systemic conditions, in addition to perinatal factors such as prematurity and low birth weight (LBW) [3,4,5,6,7]. Studies show that there is a wide variation in the timing of primary tooth eruption, with the first tooth usually expected between 6 and 10 months of age [8,9].

Prematurity has gained attention in the literature as a factor associated with delayed tooth eruption. Most studies indicate a relationship between preterm birth, defined as occurring before 37 weeks of gestation, and alterations in the dental development timeline, generally analyzed using chronological age [3,4,5,6,7]. However, few studies have accounted for ‘corrected age’, defined as the chronological age the infant would have reached had they been born at term (40 weeks of gestation) [10]. Corrected age is considered up to at least when children reach 24 months of age and is important for monitoring the growth and neurodevelopmental progress of those born preterm. Furthermore, it is used to eliminate bias and reduce the transient developmental gap of prematurity until these infants catch up with their full-term peers [11].

It is important to highlight that socioeconomic determinants have been identified as distal risk factors associated with preterm birth [12]. A higher socioeconomic status can provide greater access to prenatal consultations. These consultations are essential for assessing the pregnant woman’s health and providing appropriate guidance on how to proceed during the gestational period, including recommendations regarding the use of medications and necessary vitamin supplements [13]. Women with higher levels of education have greater access to better jobs and income, higher quality food, as well as reduced risk behaviors such as alcohol and tobacco use [14]. In addition, occupations characterized by heavy manual labor, in stressful conditions, with exposure to chemical agents or long periods at the workplace are factors that may be associated with prematurity [15].

For studying complex developmental processes like tooth eruption, which may be influenced by a range of interrelated factors, from socioeconomic conditions and maternal health during pregnancy, to gestational age, birth weight, and postnatal conditions, the development of path models is particularly useful. Path models are statistical strategies that allow for the simultaneous analysis of direct and indirect relationships among variables [16]. Therefore, the aim of this study was to test a path model to investigate the direct and indirect birth-related factors influencing the age of eruption of the first primary tooth in infants. Additionally, the study aims to assess the role of prematurity as one of the key determinants of this trajectory, given its clinical relevance and prevalence.

## 2. Materials and Methods

The present study was reported following the Strengthening the Reporting of Observational Studies in Epidemiology (STROBE statement) [16].

### 2.1. Ethical Aspects

The infants’ parents/caregivers voluntarily signed a free and informed consent form authorizing the participation of their children. This study was approved by the Human Research Ethics Committee (CAAE 66128117.4.0000.5149) and was conducted in accordance with the Declaration of Helsinki.

### 2.2. Study Design and Eligibility Criteria

This cohort study was carried out with a sample of infants of both sexes and at least four months of age. The exposed group was composed of infants who had been born preterm (before completing 37 weeks of gestational age). All preterm infants who were followed up at a multidisciplinary reference center at a Federal University Hospital in Belo Horizonte, Brazil in 2017 were invited to participate. In this center, infants born preterm had their general condition monitored monthly during the first year of life, which allowed data collection to be carried out at the same frequency.

The non-exposed group was composed of infants who had been born at term. The infants of non-exposed group were monitored monthly in private daycare centers in Belo Horizonte, Brazil. Infants with Down Syndrome, Congenital Zika Virus Syndrome, and/or with any erupted teeth were excluded from both the exposed and the non-exposed group.

### 2.3. Sample Size Calculation

The sample was calculated adopting a 95% confidence interval (95% CI) and a power of 80%, considering the mean and standard deviation of first primary tooth eruption of both preterm (9.32 ± 1.48) and full term (7.97 ± 2.62) infants by previous study [8]. The minimal sample size was 39 infants in each group, which increased by 20% to compensate for possible losses. Therefore, the final sample was estimated at 49 premature infants and 49 term infants.

### 2.4. Calibration of the Examiners

Two calibrated examiners carried out data collection. Examiner calibration was performed by analyzing photographs of the oral cavity of infants with signs of tooth eruption. The calibration was coordinated by a specialist in pediatric dentistry, considered the gold standard. The Kappa test was used to verify intra-examiner (Kappa = 0.86 and Kappa = 0.85) and inter-examiner agreement between the two examiners (Kappa = 0.87).

### 2.5. Data Collection of Observable Clinical Variables

Study participants were assessed monthly in routine appointments at the hospital and in private daycare centers. The child was no longer monitored after the tooth erupted.

Mothers responded to a questionnaire to collect the following information: the child’s sex (male/female), mother’s work during pregnancy (yes/no), medication use during pregnancy (yes/no), prenatal consultations (up to 6 consultations/7 or more consultations) [17] and whether or not the infant was the first child (yes/no).

For the preterm group, the data about the child’s health were collected in the medical records: whether the birth had had any complications (yes/no) and birth weight (extreme low birth weight < 1000 g, very low birth weight ≥ 1000 to <1500 g, low birth weight ≥ 1500 to <2500 g normal weight ≥ 2500). For the term group, the same data were collected through questionnaires for the mothers, who were advised to consult the Child Health Handbook. It is a booklet provided to families upon the child’s hospital discharge and used by both families and healthcare professionals. This handbook allows them to monitor child’s health, growth, and development from birth to age 9, as well as their childhood vaccination status, among other essential steps for comprehensive care and protection of their child’s health [18].

Oral clinical examinations were performed monthly through palpation and visual inspection of the oral cavity to detect the eruption of the first primary tooth. During oral examinations, sterile gauze was used to gently separate the soft tissues and allow observation of the oral structures, in addition to personal protective equipment. Protective stabilization during the oral examination was ensured by positioning infants from daycare centers on the caregiver’s lap and infants from the multidisciplinary reference center on the parent’s lap. The examiners used personal protective equipment. A tooth was considered erupted when any part of its crown passed through the gums and was visible in the oral cavity. Caregivers were additionally queried about the date of tooth eruption in the oral cavity to ensure enhanced control during the monthly monitoring of infants. The age of tooth eruption was considered in two forms: chronological age and corrected age. The “Chronological age” refers to the actual age of infants, calculated from the date of their birth. The term “corrected age” is used in the context of preterm infants. It represents the adjusted age of preterm infants, taking into account the time they were born prematurely, and reflects the age the infant would have been if they had been born at 40 weeks [9].

### 2.6. Observable Non-Clinical Variables

#### 2.6.1. Socioeconomic Component

The socioeconomic component was measured using the family’s income and the mother’s schooling. The family’s income was collected according to the Brazilian monthly minimum wage (R$1412), which corresponds to $286. The mother’s schooling was collected according to the grades of education (elementary school, middle school, high school, college or university).

#### 2.6.2. Infant’s Hospitalization Component

Infant’s hospitalization component was measured by the presence or the absence of each infant in the intensive care unit (ICU) and incubator, as well as the use, or not, of an endotracheal tube by the orotracheal route.

### 2.7. Statistical Analysis

The IBM SPSS Statistics (SPSS for Windows, version 26.0, IBM Inc., Armonk, NY, USA) was used for performing descriptive analyses, the Little’s test, and the Principal Component Analysis (PCA). The Little’s test was used to determine whether missing data were completely random, allowing data imputation, through expected maximization. The PCA was conducted to build formative models for the following components: the socioeconomic status and the infant’s hospitalization. The adequacy of the components was evaluated by Kaiser-Meyer-Olkin (KMO) > 0.50 and Bartlett’s test of sphericity *p* < 0.05. Factor extraction was based on the percentage of explained variance > 60% and Eigenvalue > 1. Factor loadings > 0.40 were considered satisfactory [19].

A Path Analysis was performed using Mplus software (version 8.6, Muthen & Muthen, Los Angeles, CA, USA). The Weighted Least Squares Mean and the Variance-adjusted (WLSMV) estimation method were used, since the model has categorical data [20]. Initially, two models were built: a model using the chronological age of tooth eruption and a model using the corrected age of tooth eruption. Then, an exploratory refinement procedure was conducted to improve model parsimony. Paths showing very weak associations (*p* ≥ 0.40) were removed to eliminate paths with minimal contribution to structural relationships. This approach has been adopted in path analysis contexts to reduce model complexity and retain only theoretically meaningful associations [21]. The following goodness-of-fit indices were used to evaluate the models: Comparative Fit Index (CFI), Tucker–Lewis Index (TLI), Standardized Root Mean Residual (SRMR), and Root Mean Square Error of Approximation (RMSEA). CFI and TLI values were to have been >0.90. SRMR and RMSEA values were to have been <0.08 [19].

## 3. Results

**Figure A1:**
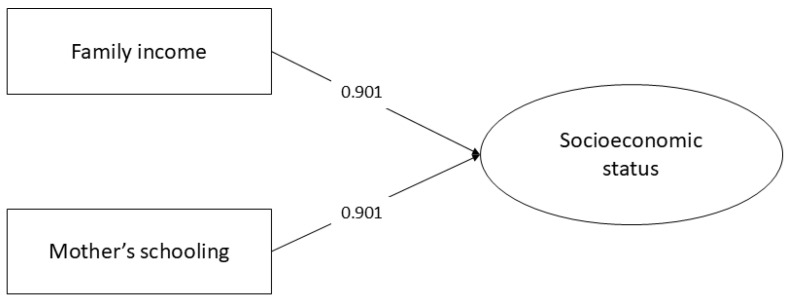
The structure and factor loadings of the formative model of socioeconomic status.

**Figure A2:**
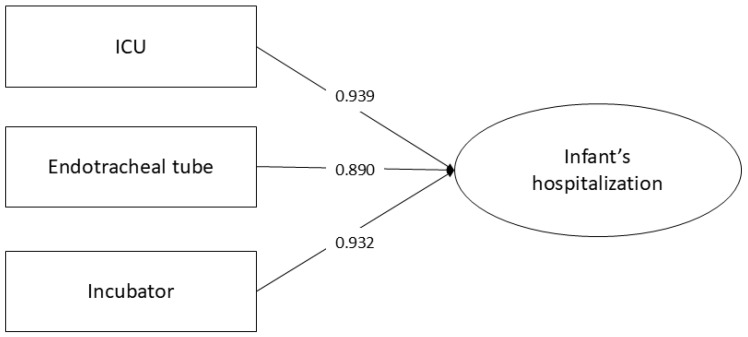
The structure and factor loadings of the formative model of the infant’s hospitalization.

Ninety-one infants participated in this study: 48 born at term and 43 infants born preterm, with 5.5% being extremely preterm (less than 28 weeks), 26.4% preterm (28 to less than 32 weeks), and 15.4% moderate to late preterm (32 to less than 37 weeks). There were no losses during the follow-up period. The sample exhibited a statistical power of 100% when examining the comparison of mean chronological age at the first tooth eruption between term and preterm children. The power for the comparison based on the corrected age stood at 99.6%. For the term-born infants, the mean age of the first tooth eruption was 7 months (±1.272), and for the preterm infants, it was 11.37 months (±2.51). When considering corrected age, the mean was 8.95 months (±2.51). Additional data are presented in Table 1. The assumptions for the PCA were met by both components: socioeconomic status component (Figure A1) and infant’s hospitalization component (Figure A2).

**Figure 1:**
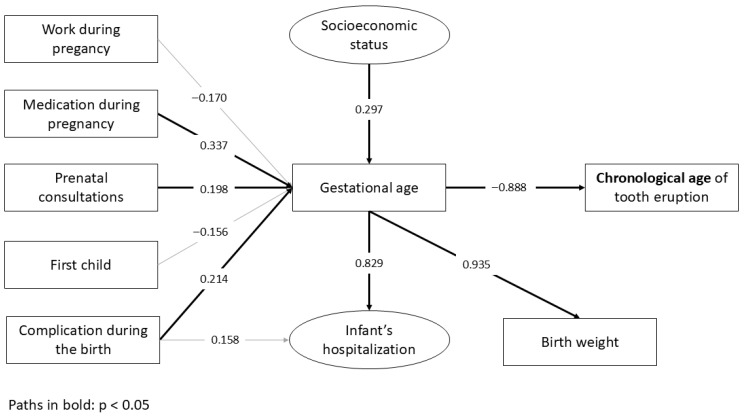
The path model with standardized coefficients (β) for the chronological age of tooth eruption.

**Figure 2:**
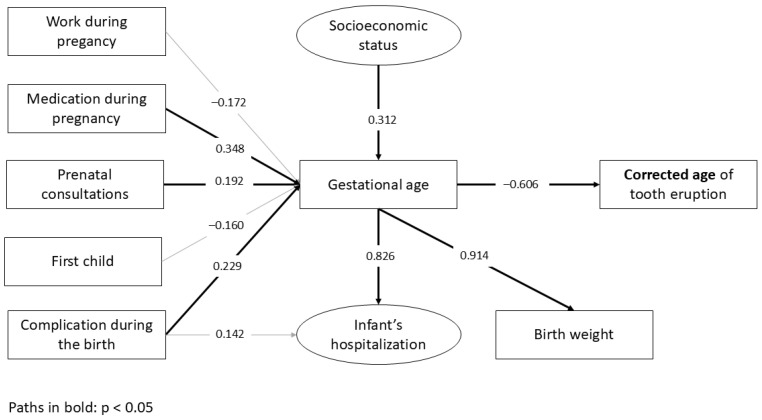
The path model with standardized coefficients (β) for the corrected age of tooth eruption.

The structural model demonstrated an almost perfect overall fit, suggesting its plausibility. In the final model of chronological age (Figure 1; Table 2), preterm infants have an increased risk of presenting a delay in tooth eruption compared to those born at term (β = −0.888; *p* < 0.001). Indirect associations were found between socioeconomic status (β = −0.264; *p* < 0.001), medication during pregnancy (β = −0.299; *p* < 0.001), ≤6 prenatal consultations (β = −0.174; *p* < 0.001) and complications during pregnancy (β = −0.190; *p* < 0.001) with chronological age of tooth eruption, which were mediated by the gestational age. Gestational age exerted a significant influence on infant’s hospitalization (β = 0.829; *p* < 0.001) and birth weight (β = 0.935; *p* < 0.001). The same direct and indirect associations found for the chronological age were demonstrated for the corrected age, with differences only in β values (Figure 2; Table 3).

## 4. Discussion

This study presents a plausible model that demonstrates the direct and indirect pathways between birth-related factors and late tooth eruption. It was observed that preterm infants, regardless of whether chronological or corrected age is used, exhibited a higher risk of later tooth eruption compared to full-term infants. Furthermore, lower socioeconomic status, medication use during pregnancy, fewer prenatal medical visits, and complications during delivery were risk factors for late tooth eruption, mediated by gestational age.

Previous studies with traditional analyses also found that preterm infants are at greater risk of later tooth eruption compared to full-term infants [3,4,5,6,7], but this is the first study to present a path model. In this way, it became possible to test multiple dependency relationships simultaneously, enabling the evaluation of a broader model. Moreover, this approach accounted for measurement error, thereby increasing the reliability of the estimated associations.

The present study demonstrated an association even when the comparison was made with the corrected age of the preterm infant, which was another novelty in relation to other studies [3,4,5,6,7]. The utilization of corrected age improves the capacity to correctly identify authentic delays, distinguishing them from perceived delays attributed to a child’s gestational age at birth [22]. Professionals must utilize corrected age instead of chronological age to make interpretations regarding the adequacy of a child’s growth or developmental progress, thereby potentially influencing the delivery of care [23].

Although birth weight and infant hospitalization are recognized in the literature as important predictors of outcomes in child development, including the formation of dental tissues and craniofacial bone structures [3,4,5,6,7], in the present study both variables were excluded from the final analysis aiming for parsimony in the model. Studies have indicated that low birth weight is related to systemic immaturity and increased vulnerability to metabolic and nutritional disturbances, which may interfere with the chronology of primary tooth eruption [3,4,5,6,7]. In the present study, gestational age significantly influenced both neonatal hospitalization and birth weight, highlighting the interconnection among these variables. Therefore, birth weight should be considered a relevant marker for the early screening of risks to infant oral development.

Regarding complications during childbirth, the literature shows that risks to maternal health, such as teenage pregnancy, infections, malnutrition and pre-eclampsia, are closely linked to premature births [10]. Furthermore, those complications may be related to the health conditions of premature infants, who are more vulnerable to adverse conditions compared to full-term infants. Pregnant women’s health is a political and social issue. The Brazilian Ministry of Health determines at least six prenatal follow-up consultations, preferably one in the first trimester, two in the second, and three in the third trimester of pregnancy [17]. Health policies are necessary to disseminate information related to the topic to pregnant women who do not understand the importance of good prenatal care [10].

This study has some limitations. It is not population-based, and only the eruption of the first primary tooth was evaluated. It is still necessary to carry out a representative study and a cohort that monitors the eruption of all teeth. Furthermore, the recruitment of full-term infants from private daycare centers and preterm infants from a public multidisciplinary reference center may have introduced socioeconomic differences between the groups. Although this strategy was adopted for reasons of feasibility and access to the target population, we recognize that socioeconomic factors may have influenced the results and acted as potential confounders. Future studies should consider recruitment strategies that ensure greater socioeconomic comparability between groups in order to minimize this type of bias. It is also important to highlight that, for the full-term group, data on the child’s health was obtained through the Child Health Handbook. However, this source is prone to registration errors and variability in information quality, representing a limitation to data accuracy when compared to medical chart records, which were used for preterm group. Nevertheless, the present study was the first to incorporate a complex model, with multiple dependency relationships, to better understand the connection between gestational factors, premature birth, and corrected and chronological age.

This study revealed important findings for clinical practice and highlights the need for greater attention to the oral health of newborns, particularly those born prematurely, as they may be at greater risk of oral development disorders. A delayed tooth eruption may indicate developmental issues and often causes considerable anxiety in parents. Uncertainty about whether their child is developing normally can lead to increased emotional stress, especially in families already facing challenges related to prematurity. For many parents, absent or delayed tooth eruption may be perceived as a visible marker of abnormality, leading to frequent consultations with healthcare professionals [24]. Therefore, pediatric dentists must be prepared to provide clear and evidence-based guidance. This highlights the importance of including anticipatory guidance on tooth eruption schedules as part of routine pediatric and neonatal dental care.

It is important to highlight that despite regular consultations with pediatricians being common practice, the same cannot be said about evaluations with pediatric dentists [25]. Hence, it would be beneficial for pediatricians to understand the significance of referring infants to pediatric dentists, enabling them to effectively guide parents. Furthermore, the study reinforces the value of public maternal health policies that aim to improve access to quality prenatal care, particularly for mothers from lower socioeconomic backgrounds, as a means of preventing complications during pregnancy and childbirth. Finally, the results emphasize the relevance of using corrected age in epidemiological studies, since delayed tooth eruption has been observed in preterm newborns even after adjustment for corrected age.

## 5. Conclusions

It was concluded that the preterm infants exhibited a higher risk of delayed tooth eruption compared to full-term infants, considering both chronological and corrected age. Gestational age mediated the association between socioeconomic status, medication use during pregnancy, prenatal visits, and birth complications with the age of tooth eruption.

## Figures and Tables

**Table 1:** Descritive data of the studied groups (n = 91).

Variables	At Term	Preterm
	N (%)	N (%)
**Infant’s demographic characteristics**		
Sex		
Female	25 (52.1)	22 (51.2)
Male	23 (47.9)	21 (48.8)
Infant’s health		
Birth weight		
Low weight	2 (4.2)	23 (53.5)
Normal weight	46 (95.8)	20 (46.6)
Stayed in the ICU		
Yes	0 (0)	39 (90.7)
No	48 (100)	4 (9.3)
Endotracheal tube use		
Yes	0 (0)	29 (67.4)
No	48 (100)	14 (32.6)
Stayed in the incubator		
Yes	1 (2.1)	39 (90.7)
No	47 (97.9)	4 (9.3)
**Mother’s health**		
Worked during pregnancy		
Yes	40 (83.3)	25 (58.1)
No	8 (16.7)	17 (39.5)
Medication during pregnancy ^†^		
Yes	22 (54.8)	22 (54.8)
No	26 (54.2)	26 (54.2)
Prenatal consultations		
Up to 6 consultations	8 (16.7)	22 (51.2)
7 or more queries	39 (81.3)	21 (48.8)
Complication during pregnancy ^‡^		
Yes	1 (2.1)	13 (30.2)
No	47 (97.9)	30 (69.8)
First child		
Yes	33 (68.8)	20 (46.5)
No	15 (31.3)	23 (53.5)
**Mother’s demographic characteristics**		
Mother’s education		
Up to 11 years of studies	16 (33.3)	36 (83.7)
More than 11 years of studies	31 (64.6)	7 (16.3)
Income		
Up to 2 minimum wages	17 (35.4)	28 (65.1)
Above 3 minimum wages	31 (64.6)	11 (25.6)

Notes: ICU: intensive care unit ^†^ Medication during pregnancy: hormones, vitamins and supplements, antibiotics, anti-inflammatories, corticosteroids, anti-diabetic drugs, antihypertensive and heart drugs, antidepressants, antiretroviral therapy drugs. ^‡^ Complication during pregnancy: fetal distress, hemorrhage, eclampsia, pre-eclampsia, obstetric violence, infection. Some variables exhibited a loss, resulting in the total less than 100%.

**Table 2:** Standardized Coefficients of Structural Equation Modeling (Chronological age of tooth eruption).

Ways	Initial Model Standardized Coefficients (*p*-Value)	Final Model Standardized Coefficients (*p*-Value)	Confidence Interval	Standard Error
**Gestational age ON**				
Socioeconomic status	0.295 (0.011)	0.297 (0.010)	0.107–0.487	2.57
Work during pregnancy	−0.169 (0.066)	−0.170 (0.068)	−0.324–−0.017	−1.82
Medication during pregnancy	0.335 (<0.001)	0.337 (<0.001)	0.183–0.490	3.61
Prenatal consultations	0.197 (0.026)	0.198 (0.025)	0.053–0.343	2.25
Number of births	−0.155 (0.072)	−0.156 (0.070)	−0.298–−0.014	−1.81
Complication during the birth	0.225 (0.031)	0.214 (0.007)	0.084–0.345	2.70
**Infant’s hospitalization ON**				
Gestational age	0.834 (<0.001)	0.829 (<0.001)	0.749–0.909	17.0
Complication during the birth	0.148 (0.073)	0.158 (0.080)	−0.009–0.307	1.75
**Birth weight ON**				
Gestational age	0.934 (<0.001)	0.935 (<0.001)	0.823–1.048	13.6
**Chronological age of tooth eruption ON**				
Gestational age	−1.305 (0.024)	−0.888 (<0.001)	−0.979–−0.797	−16.0
Birth weight	0.438 (0.448)	-	-	-
Complication during the birth	0.018 (0.854)	-	-	-
**General Fit Indices**				
CFI	0.999	1.000	-	-
TLI	0.998	1.000	-	-
SRMR	0.094	0.095	-	-
RMSEA (IC95%)	0.010 (0.000–0.096)	0.001 (0.001–0.093)	-	-

CFI, comparative fit index; TLI, Tucker–Lewis Index; RMSEA, root mean square error of approximation; SRMR, standardized root mean square residual; IC, confidence interval.

**Table 3:** Standardized Coefficients of Structural Equation Modeling (Corrected age of tooth eruption).

Ways	Initial Model Standardized Coefficients (*p*-Value)	Final Model Standardized Coefficients (*p*-Value)	Confidence Interval	Standard Error
**Gestational age ON**				
Socioeconomic status	0.309 (0.010)	0.312 (0.010)	0.113–0.512	2.57
Work during pregnancy	−0.170 (0.076)	−0.172 (0.075)	−0.332–−0.013	−1.78
Medication during pregnancy	0.345 (<0.001)	0.348 (<0.001)	0.188–0.508	3.58
Prenatal consultations	0.190 (0.040)	0.192 (0.040)	0.038–0.345	2.06
Number of births	−0.159 (0.064)	−0.160 (0.065)	−0.303–−0.018	−1.85
Complication during the birth	0.221 (0.031)	0.229 (0.013)	0.078–0.380	2.49
**Infant’s hospitalization ON**				
Gestational age	0.832 (<0.001)	0.826 (<0.001)	0.743–0.909	16.42
Complication during the birth	0.148 (0.070)	0.142 (0.121)	−0.009–0.293	1.55
**Birth weight ON**				
Gestational age	0.935 (<0.001)	0.914 (<0.001)	0.811–1.017	14.56
**Chronological age of tooth eruption ON**				
Gestational age	−1.164 (0.092)	−0.606 (<0.001)	−0.785–−0.428	−5.58
Birth weight	0.599 (0.374)	-	-	-
Complication during the birth	−0.007 (0.949)	-	-	-
**General Fit Indices**				
CFI	1.000	1.000	-	-
TLI	1.000	1.000	-	-
SRMR	0.104	0.100	-	-
RMSEA (IC95%)	0.000 (0.000–0.094)	0.000 (0.000–0.089)	-	-

CFI, comparative fit index; TLI, Tucker–Lewis Index; RMSEA, root mean square error of approximation; SRMR, standardized root mean square residual; IC, confidence interval.

## Data Availability

Dataset available on request from the authors.

## Abbreviations

The following abbreviations are used in this manuscript:

LBW	Low birth weight
STROBE	Strengthening the Reporting of Observational Studies in Epidemiology
CI	Confidence interval
ICU	Care unit
KMO	Kaiser-Meyer-Olkin
WLSMV	Weighted Least Squares Mean and the Variance-adjusted
CFI	Comparative Fit Index
TLI	Tucker–Lewis Index
SRMR	Standardized Root Mean Residual
RMSEA	Root Mean Square Error of Approximation
